# Fragmented QRS on far‐field intracardiac electrograms as a predictor of arrhythmic events

**DOI:** 10.1002/joa3.12622

**Published:** 2021-08-21

**Authors:** Yuki Osaka, Yuichi Ono, Kentaro Goto, Kento Yabe, Akifumi Tanaka, Toru Miyazaki, Asami Suzuki, Ken Kurihara, Masahiko Goya, Kenichiro Otomo, Tetsuo Sasano

**Affiliations:** ^1^ Department of Cardiology Ome Municipal General Hospital Tokyo Japan; ^2^ Department of Cardiovascular Medicine Tokyo Medical and Dental University Tokyo Japan

**Keywords:** arrhythmic event, fragmented QRS, implantable cardioverter‐defibrillator

## Abstract

**Background:**

Studies suggest that fragmented QRS (fQRS) can predict arrhythmic events in various cardiac diseases. However, the association between fQRS recordings on intracardiac electrogram (EGM) and ventricular arrhythmic events remains unknown.

**Methods:**

We enrolled 51 patients (age, 62 ± 12 years; 40 men) with an implantable cardioverter‐defibrillator (ICD) and structural heart disease and evaluated surface electrocardiogram (ECG) and EGM measurement of fQRS and the association between fQRS and arrhythmic events.

**Results:**

fQRS was detected on surface ECG and ICD‐EGM in 12 (23.5%) and 15 (29.4%) patients, respectively. fQRS was detected more frequently on ICD‐EGM in patients with fQRS on surface ECG than in patients without fQRS (7/12 [58.3%] vs 8/39 patients [20.5%], *P* = .01). Appropriate ICD therapies were documented in 16 patients. Among these patients, fQRS was detected more frequently on surface ECG and ICD‐EGM in patients with appropriate ICD therapies (8/16, 50.0%; *P* = .001 and 11/16, 68.9%; *P* < .001). Nonsustained ventricular tachycardia was significantly more frequent in patients with appropriate ICD therapies (15/16, 93.8%; *P* = .04). Multiple logistic regression analysis showed that fQRS on ICD‐EGM was a predictor of arrhythmic events (*P* = .03). Kaplan‐Meier survival analysis revealed that ICD therapies were significantly more frequent among patients with fQRS on both surface ECG and ICD‐EGM than among those without fQRS (66.7% vs 6.6%, *P* < .001).

**Conclusion:**

The presence of fQRS on ICD‐EGM can be a predictor of arrhythmic events in ICD patients. Surface ECG and ICD‐EGM measurement may help predict ventricular arrhythmic events.

## Graphical abstract

We enrolled patients with implantable cardioverter defibrillator (ICD) and structural heart disease and evaluated the surface electrocardiogram (ECG) and intracardiac electrogram (EGM) measurement of fQRS and the association between fQRS and arrhythmic events.Appropriate ICD therapies were significantly higher in patients with fQRS on both surface ECG and ICD EGM than in those without.Surface ECG and ICD‐EGM measurement may help predict ventricular arrhythmic events.

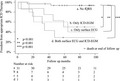

## 1 INTRODUCTION

Fragmented QRS (fQRS) represents a conduction delay caused by myocardial scarring in patients with coronary artery disease (CAD).^1^, ^2^ However, fQRS is not specific to CAD and is also encountered in other myocardial diseases, such as cardiomyopathy and congenital heart disease.^3^, ^4^, ^5^, ^6^, ^7^, ^8^, ^9^, ^10^, ^11^, ^12^, ^13^, ^14^, ^15^, ^16^, ^17^ It has been reported that fQRS can predict ventricular arrhythmic events in various cardiac diseases.^2^, ^6^, ^7^, ^8^, ^11^, ^12^, ^13^, ^18^, ^19^

The association between fQRS recordings on intracardiac electrogram (EGM) and ventricular arrhythmic events in patients with an implantable cardioverter‐defibrillator (ICD) remains unknown. This study aimed to evaluate the association between fQRS recordings on the surface electrocardiogram (ECG) and intracardiac EGM in patients with ICD (ICD‐EGM) and appropriate ICD therapies, defined as shock and antitachycardia pacing (ATP) for ventricular arrhythmia.

## 2 METHODS

### 2.1 Study population

In this retrospective study, we investigated 51 patients with ICD and structural heart disease, excluding patients with hereditary arrhythmia disease, between January 2003 and May 2016. Patients were followed up every 4 months at the ICD Clinic until January 2020. The outcome of this study was appropriate ICD therapy. We evaluated surface ECG, ICD‐EGM, and appropriate ICD therapies for all patients. Surface ECG and ICD‐EGM were evaluated without information on ICD treatment and surface ECG or ICD‐EGM. This study was approved by our respective institutional review boards. Informed consent was obtained in the form of opt‐out on the website.

### 2.2 ECG criteria for fQRS (RSR pattern and its variants)

fQRS included the presence of an additional R wave (R) or notching in the nadir of the R wave or the S wave, or the presence of more than one R (fragmentation) in two contiguous leads, corresponding to a major coronary artery territory.^1^ If the RSR patterns were present in the right precordial leads (leads V1 and V2) with QRS of >100 ms (incomplete right bundle branch block) or QRS of >120 ms (complete right bundle branch block) and in the left precordial lead (RSR in leads I, V5, and V6) with QRS of >120 ms (left bundle branch block), this was defined as a complete or incomplete bundle branch block and was excluded from the definition of fQRS. If the RSR pattern was present in the mid‐precordial or inferior leads, this was defined as fQRS. A low‐pass filter is frequently used to reduce electrical and muscular noise when recording the 12‐lead ECG; however, the cutoff frequency of the low‐pass filter influences the detection of fQRS.^20^ In our study, ECGs were recorded using a low‐pass filter at 150 Hz. Bandpass filters for ICD‐EGM vary by manufacturer and their settings are not published.

Near‐field (NF) EGM was defined as the difference in potentials between the tip and the ring, or between the tip and the coil of the bipolar ICD lead implanted in the apex of the right ventricle (RV). Far‐field (FF) EGM was defined as the difference in potentials between the ring of the RV lead and the ICD can, or between the coil and can. The fQRS of ICD‐EGM was reported if it was recorded by either lead.

### 2.3 Appropriate ICD therapy

Appropriate ICD therapy was defined as shock and ATP for ventricular arrhythmia. ICD programming for ventricular tachyarrhythmia was based on the judgment of the attending physician.

### 2.4 Statistical analysis

Continuous variables are expressed as mean ± standard deviation. Continuous and categorical variables were compared using Student's t‐test and the chi‐square test, respectively. A Cox proportional hazard model was used to assess the association of the baseline variables with the appropriate ICD therapy. Survival curves were calculated using the Kaplan‐Meier method. *P*‐values of <.05 were considered statistically significant.

All data were analyzed using PASW 18 software (SPSS Inc). A *P*‐value of <.05 was considered statistically significant.

## 3 RESULTS

### 3.1 Patient characteristics

We investigated 51 patients (age, 62 ± 12 years; 40 male patients) with ICD (n = 35, Medtronic; n = 12, St. Jude Medical; n = 4, Boston Scientific) and structural heart diseases. The baseline characteristics of the patients are shown in Table 1. Twenty‐four (47.1%), 9 (17.6%), 5 (9.8%), and 3 (5.9%) patients had ischemic heart disease, hypertrophic cardiomyopathy (HCM), dilated cardiomyopathy (DCM), and arrhythmogenic right ventricular cardiomyopathy (ARVC), respectively.

**TABLE 1:** Patients characteristics

	n = 51
Age (years)	62.0 ± 12.3
Male (%)	40 (78.4)
Hypertension (%)	17 (33.3)
Diabetes mellitus (%)	9 (17.6)
Hyperlipidemia (%)	28 (54.9)
Chronic kidney disease (%)	20 (39.2)
Ejection fraction (%)	51.3 ± 16.9
Secondary prevention (%)	15 (29.4)
NSVT (%)	34 (66.7)
EPS (positive/underwent)	35/38
QRS duration (ms)	111 ± 28.1
fQRS on surface ECG (%)	12 (23.5)
fQRS on intracardiac EGM (%)	15 (29.4)
Underlying heart disease (%)	
IHD	24 (47.1)
HCM	9 (17.6)
DCM	5 (9.8)
ARVC	3 (5.9)
others	10 (19.6)
Drug therapy (%)	
β‐blocker	37 (72.5)
ACEI/ARB	27 (52.9)
Amiodarone	26 (52.9)
Sotalol	1 (2.0.0)

Note

Data are presented as the n (%) or mean ± SD.

Abbreviations: ACEI, Angiotensin converting enzyme inhibitor; ARB, Angiotensin II receptor blocker; ARVC, Arrhythmogenic right ventricular Cardiomyopathy; DCM, Dilated cardiomyopathy; EPS, Electrophysiological study; fQRS, fragmented QRS; HCM, Hypertrophic cardiomyopathy; IHD, Ischemic heart disease; NSVT, Nonsustained ventricular tachycardia.

### 3.2 Detection of fQRS

**FIGURE 1:**
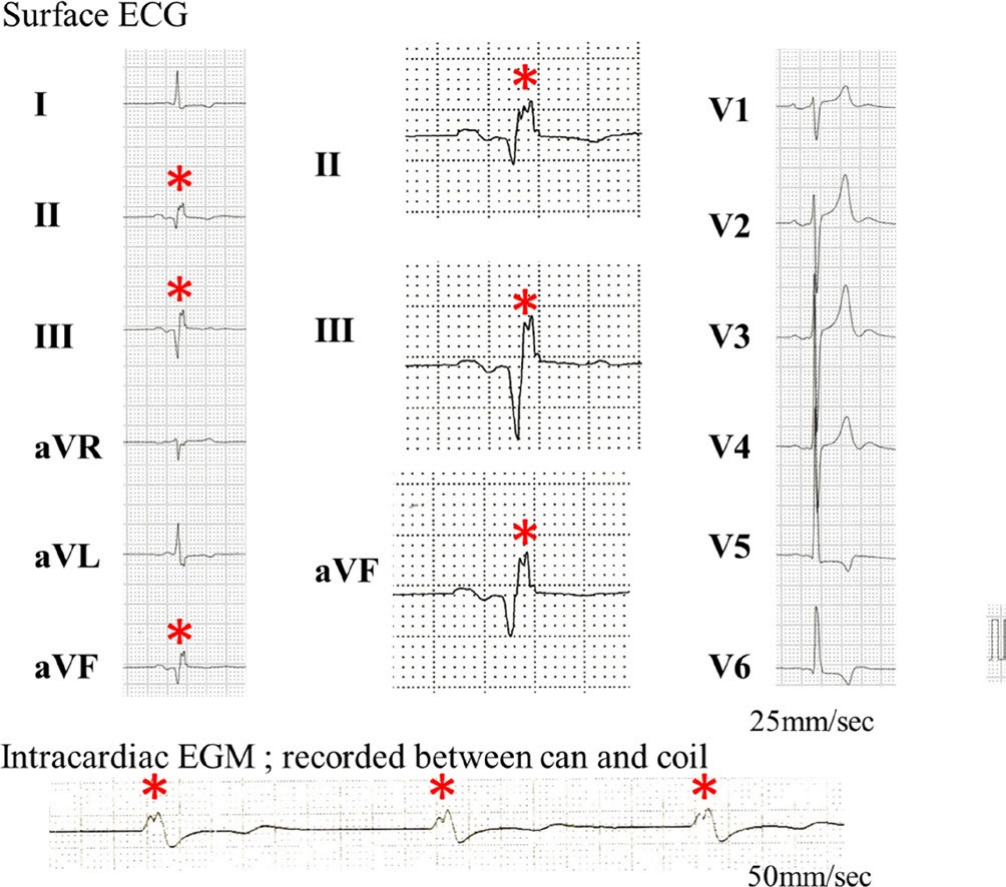
Twelve‐lead electrocardiography shows an RsR' pattern in inferior leads. Fragmented QRS were recorded on a far‐field electrocardiogram (between the can and coil)

fQRS on surface ECG was detected in 12 (23.5%) patients, fQRS on surface ECG was recorded on inferior leads in all patients, and fQRS on the ICD‐EGM was recorded in 15 (29.4%) patients. fQRS was recorded more frequently on FF EGM than on NF EGM (15/15 patients, 100% vs 5/15 patients, 33.3%). Recordings were as follows: between the can and coil, 15 patients; between the can and ring, 3 patients; between the tip and coil, 3 patients; and between the tip and ring, 3 patients. Patients with fQRS recorded on NF EGM also had fQRS on FF EGM. Only 1 patient presented fQRS on all polarities. The cases of fQRS on surface ECG and ICD‐EGM are shown in Figure 1. fQRS on ICD‐EGM was recorded in 7 out of 12 patients with fQRS on surface ECG (Table 2).

**TABLE 2:** Fragmented QRS

		Surface ECG	
		fQRS (+)	fQRS(−)
ICD‐EGM	fQRS (+)	7	8
	fQRS (−)	5	31

Abbreviations: ECG, Electrocardiogtaphy; EGM, Electrocardiogram; fQRS, fragmented QRS; ICD: implantable cardioverter defibrillator.

### 3.3 Appropriate ICD therapy

During follow‐up, appropriate ICD therapies were observed in 16 patients (Table 3). fQRS on surface ECG and ICD‐EGM was significantly more frequent in patients with appropriate ICD therapies than in those without (8/16 [50.0%; *P* = .001] and 11/16 [68.9%; *P* < .001]) (Table 3).

**TABLE 3:** Appropriate ICD therapy

	ICD therapy (+) n = 16	ICD therapy(−) n = 35	*P* value	Multivariate *P* value	OR (95% CI)
Age, years	60.5 ± 9.33	62.6 ± 13.5	.51		
Male (%)	15 (93.8)	25 (71.4)	.06		
Hypertension	9 (56.3)	8 (22.9)	.03	.07	3.01 (0.90‐10.1)
Diabetes mellitus	2 (12.5)	7 (20.0)	.65		
Hyperlipidemia	6 (37.5)	22 (62.9)	.06		
Chronic kidney disease	8 (50.0)	12 (34.3)	.20		
Ejection fraction (%)	46.9 ± 18.4	53.3 ± 16.0	.12		
QRS duration (%)	125 ± 30.8	104 ± 23.8	.008	.07	1.02 (0.99‐1.04)
IHD (%)	5 (31.3)	19 (54.3)	.23		
Secondary prevention	1 (6.3)	14 (40.0)	.05		
NSVT (%)	15 (93.8)	19 (54.3)	.04	.44	2.43 (0.25‐23.8)
fQRS on surface ECG (%)	8 (50.0)	4 (11.4)	.001	.25	2.02 (0.61‐6.65)
fQRS on ICD‐EGM (%)	11 (68.9)	4 (11.4)	<.001	.03	3.78 (1.14‐12.6)

Note

Data are presented as the n (%) or mean ± SD.

Abbreviations: ECG, Electrocardiography; EGM, Electrocardiogram; fQRS, fragmented QRS; ICD, implantable cardioverter defibrillator; IHD, ischemic heart disease; NSVT, nonsustained ventricular tachycardia.

Multiple logistic regression analysis showed that fQRS on ICD‐EGM was a predictor of arrhythmic events (*P* = .03) (Table 3).

fQRS on ICD‐EGM was more frequently recorded in patients with fQRS on surface ECG than in those without fQRS on surface ECG (7/12 [58.3%] vs 8/39 [20.5%], *P* = .01) (Table 2).

**FIGURE 2:**
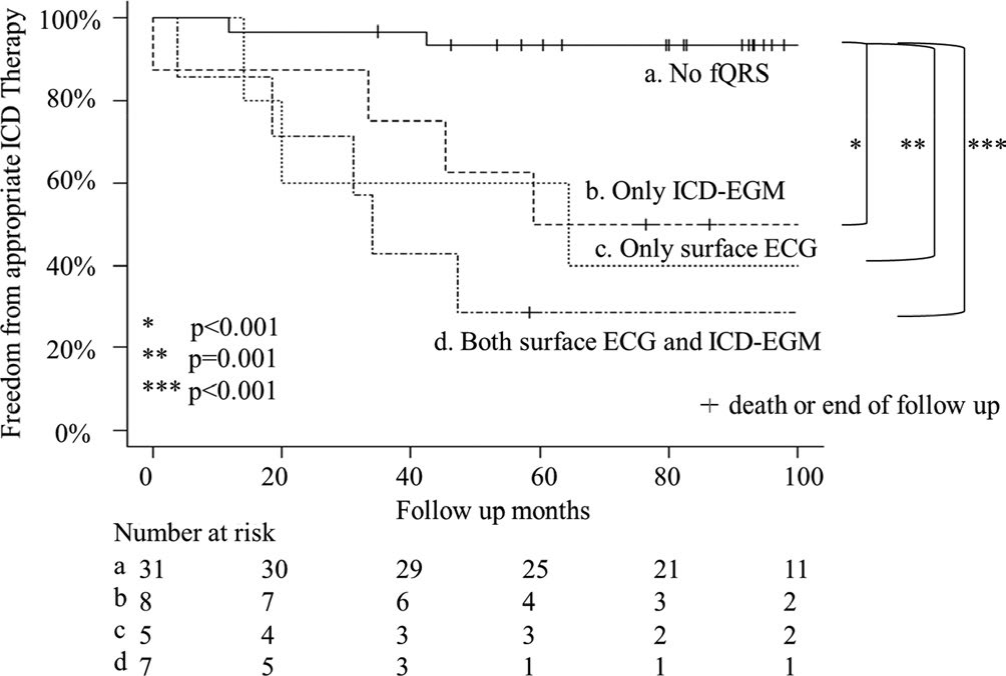
Freedom from appropriate ICD therapy: appropriate ICD therapies are significantly higher in patients with fQRS on both surface ECG and ICD‐EGM than in those without

Kaplan‐Meier survival analysis revealed that patients with fQRS on both surface ECG and ICD‐EGM had a significantly higher rate of appropriate ICD therapies than those without fQRS (66.7% vs 6.6%, *P* < .001) (Figure 2). Recording fQRS on both surface ECG and ICD‐EGM had a high positive predictive value (PPV; 85.7%) for appropriate ICD therapies. The sensitivity, specificity, and negative predictive value (NPV) for appropriate ICD therapies on recording fQRS using both surface ECG and ICD‐EGM were 37.5%, 97.1%, and 77.3%, respectively.

## 4 DISCUSSION

### 4.1 Main findings

This study is the first to evaluate the association between surface ECG, ICD‐EGM, and appropriate ICD therapies. We suggest that evaluation using surface ECG in combination with ICD‐EGM is a strong predictor of ventricular arrhythmic events. Appropriate ICD therapies were significantly more common in patients with fQRS on both surface ECG and ICD‐EGM than in those without fQRS.

### 4.2 Appropriate ICD therapy

During follow‐up, appropriate ICD therapies were observed for 16 patients. ICD programming was determined by the judgment of the attending physician. This is a limitation of our study. Seven patients programmed ATP therapy for fast ventricular tachycardia (VT) zone (240‐300 ms). Twelve patients were not programmed ATP therapy for VT but were programmed only therapy for ventricular fibrillation. No significant differences concerning these management options between patients with appropriate ICD therapy and those without appropriate ICD therapy were observed.

### 4.3 Association between fQRS and arrhythmic events

fQRS has been shown to be associated with increased mortality and arrhythmic events in patients with CAD,^1^, ^2^, ^6^, ^9^ Brugada syndrome, and nonischemic cardiomyopathy.^2^, ^6^, ^7^, ^8^, ^11^, ^12^, ^13^, ^17^, ^18^ Theories of the mechanism of fQRS on the routine 12‐lead ECG are speculative. Fragmentation of QRS has been implicated in the heterogeneous activation of the ventricles due to myocardial ischemia, scarring, and/or fibrosis, which predict arrhythmic events, as well as death.^18^, ^21^ In DCM, the scars are patchy and mid‐myocardial and located predominantly in the perivalvular areas of the ventricles. In patients with DCM, HCM, and cardiac sarcoidosis, fQRS has been shown to signify myocardial scarring or involvement defined by gadolinium delayed enhancement on cardiac magnetic resonance imaging.^10^, ^14^, ^22^ Endocardial and epicardial mapping in patients with CAD and DCM with ventricular arrhythmias have revealed fractionated ECGs.^8^ Increased myocardial fibrosis in patients with HCM not only results in left ventricular systolic dysfunction and heart failure but may also provide further structural substrates for arrhythmogenicity.^22^ fQRS in sarcoidosis probably represents active inflammatory lesions and more advanced myocardial damage.^14^, ^15^ Different morphologies of fQRS are caused by the shifting of the QRS vector during depolarization in and around the areas of scarring or ischemic myocardium, depending on their extent and location in the ventricles. Altered conduction patterns result in slow conduction in the myocardial scar border zones, which may promote reentry and malignant ventricular arrhythmias.^8^

The underlying heart disease of patients is shown in Table 1. Patients with ICD had relatively preserved ejection fraction (EF), despite the rate of patients who received ICD for primary prevention being 70.6%. The underlying heart diseases of patients with ICD who have relatively preserved EF included HCM and ARVC. Regarding HCM patients, they have major risk factors, syncope or nonsustained VT (NSVT), and left ventricle outflow tract obstruction. ARVC patients exhibit an electrophysiological study (EPS)‐induced ventricular tachycardia or spontaneous stable hemodynamic VT or NSVT. The ICD indications of our study patients also included those that were not of Class I according to the Japanese nonpharmacological guidelines.^23^ These risk factors are reasons for Class IIa adaptations of the ICD indications and were potential reasons explaining the relatively preserved EF and a high percentage of primary prevention among these patients.

Japanese nonpharmacological guidelines^23^ do not recommend EPS studies for primary prophylactic implantation of ICDs in patients with organic heart diseases such as ischemic or nonischemic heart disease with preserved left ventricular EF. In our study, 38/51 (74.5%) patients had undergone EPS. Among the 35 patients who had positive results on EPS, 6 presented fQRS on the surface ECG and 8 presented fQRS on the ICD‐EGM. Patients who were EPS positive do not necessarily have fQRS on surface ECG or ICD EGM. In our study, EPS was not performed in patients receiving secondary prevention or low EF. Although it cannot be determined accurately, fQRS may be more suitable for predicting ventricular arrhythmic events than EPS.

In our study, fQRS on surface ECG was recorded in inferior leads. It has been reported that patients with fQRS in the inferior leads have a poor prognosis.^9^ An ICD was implanted in all patients included in our study. These patients might have had more arrhythmogenic substrates, which led to a poor prognosis.

One limitation of our study was that the settings of the bandpass filter vary based on the manufacturers. However, we did not observe any differences in the fQRS positive rate in ICD‐EGM depending on the device manufacturer in this study. The positive rate of fQRS for each manufacturer was as follows, n = 11/35 (31.4%), Medtronic; n = 3/12 (25.0%), St. Jude Medical; n = 1/4 (25.0%), Boston Scientific.

We anticipated that we could capture a detailed local potential on ICD‐EGM. A majority of fQRS was recorded in FF EGM (between the can and coil) because FF EGM could capture a broader area of excitement conduction than NF EGM. Because the RV lead was positioned in the RV, in our speculation, the fQRS of ICD‐EGM captured the local myocardial damage of the RV or RV septal apex. In our data, fQRS on surface ECG was recorded in the inferior lead. However, ICD‐EGM also captured electrical damage not limited to the inferior area, which could be captured on surface ECG. Recording ICD‐EGM in addition to a 12‐lead surface ECG may indicate a large area of arrhythmogenic substrate. Recording fQRS on both surface ECG and ICD‐EGM had high PPV for appropriate ICD therapies. It has been reported that microvolt T wave alternans (TWA) and late potentials (LPs) identified by time‐domain analysis of the signal‐averaged ECG are a noninvasive marker for the identification of sudden cardiac death or ventricular tachyarrhythmia in patients with postmyocardial infarction or other cardiac diseases.^24^, ^25^, ^26^, ^27^, ^28^, ^29^ This index has a high NPV (>98%). The sensitivity, specificity, and PPV for sudden cardiac death (SCD) or ventricular tachyarrhythmia of TWA were indicated to be 83%‐92%, 61%‐83%, and 7%‐9%, respectively.^24^, ^25^, ^26^ Furthermore, the sensitivity, specificity, and PPV for sudden cardiac death (SCD) or ventricular tachyarrhythmia of LPs have been reported to be 48%‐90%, 60%‐89%, and 11%‐25%, respectively.^27^, ^28^, ^29^ TWA is a marker of repolarization abnormality, and LP is a marker of depolarization abnormality. Besides LPs, fQRS can reflect intracardiac conduction abnormalities and represent a substrate for ventricular arrhythmia. fQRS is also a marker of depolarization abnormality. There is a difference between the predictive values of LPs and fQRS despite both being markers of depolarization abnormality. It is unknown whether there is a correlation between fQRS and LPs. It has been reported that fQRS may exist independently from that of LPs in patients with Brugada syndrome.^20^ The report suggested that delayed activation within a small mass of ventricular tissue could produce LPs without having significant effects on the QRS complex; delayed activation in a larger ventricular mass can cause multiple spikes within the QRS complex. Disorganized ventricular depolarization depends on the spatial and temporal patterns of impulse conduction in the pathological substrate of the ventricles, and therefore, fragmentation of QRS can occur in the early, mid, and late phases of the QRS wave. This might be a reason why fQRS may or may not coexist with LPs.^19^ Although our data had some limitations, fQRS is a simple and overt ECG sign, and further research is needed to improve its predictive value of ventricular arrhythmic events. We suggest that evaluations using surface ECG in combination with ICD‐EGM as a strong predictor of ventricular arrhythmic events.

### 4.4 Limitations

First, this study was retrospective in nature, and fQRS on surface ECG and ICD‐EGM may not have been recorded in all patients at the same timepoint. Second, this was a small‐scale study with a limited number of patients. Third, the changes in fQRS caused by progressing heart failure and/or ischemic events during follow‐up were not evaluated. Fourth, the settings of the bandpass filter may vary by company, and the cutoff frequency of the low‐pass filter may influence the detection of fQRS on ICD‐EGM. Fifth, the decision for ICD management of ventricular tachyarrhythmia was determined by the judgment of the attending physician. ICD programming can affect ICD therapies.

## 5 CONCLUSION

The presence of fQRS on ICD‐EGM can be a predictor of arrhythmic events in patients with ICD. We suggest using surface ECG in combination with ICD‐EGM to predict ventricular arrhythmic events.

## CONFLICT OF INTEREST

The authors declare no conflict of interest for this article.
